# Foxp3^+^ Treg Expanded from Patients with Established Diabetes Reduce Helios Expression while Retaining Normal Function Compared to Healthy Individuals

**DOI:** 10.1371/journal.pone.0056209

**Published:** 2013-02-11

**Authors:** Weiting Du, Yueh-Wei Shen, Wen-Hui Lee, Ding Wang, Sachiko Paz, Fouad Kandeel, Chih-Pin Liu

**Affiliations:** 1 Department of Diabetes and Metabolic Diseases Research, Beckman Research Institute, City of Hope, Duarte, California, United States of America; 2 Department of Immunology, Beckman Research Institute, City of Hope, Duarte, California, United States of America; McGill University Health Center, Canada

## Abstract

Foxp3^+^ regulatory T cells (Treg) play a crucial role in regulating immune tolerance. The use of Treg to restore immune tolerance is considered an attractive novel approach to inhibit autoimmune disease, including type 1 diabetes (T1D), and to prevent rejection of organ transplants. In view of the goal of developing autologous Treg-based cell therapy for patients with long-term (>15 years) T1D, it will be necessary to expand a sufficient amount of functional Treg *in vitro* in order to study and compare Treg from T1D patients and healthy subjects. Our results have demonstrated that there is a comparable frequency of Treg in the peripheral blood lymphocytes (PBLs) of patients with long-term T1D relative to those in healthy subjects; however, Th1 cells, but not Th17 cells, were increased in the T1D patients. Further, more Treg in PBLs from T1D patients than from healthy subjects expressed the CD45RO^+^ memory cell phenotype, suggesting they were antigen-experienced cells. After isolation, Treg from both T1D patients and healthy subjects were successfully expanded with high purity. Although there was no difference in Helios expression on Treg in PBLs, *in vitro* expansion led to fewer Helios-expressing Treg from T1D patients than healthy subjects. While more Th1-like Treg expressing IFN-γ or TNF-α were found in the PBLs of T1D patients than healthy controls, there was no such difference in the expanded Treg. Importantly, expanded Treg from both subject groups were able to suppress autologous or allogeneic CD8^+^ effector T cells equally well. Our findings demonstrate that a large number of *ex vivo* expanded functional Treg can be obtained from long-term T1D patients, although fewer expanded Treg expressed a high level of Helios. Thus, based on the positive outcomes, these potent expanded Treg from diabetic human patients may be useful in treating T1D or preventing islet graft rejection.

## Introduction

Broken down of immune tolerance often leads to auto-reactive T-cell activation, which is pivotal for the development of autoimmune diseases, including Type 1 diabetes (T1D) [1], [2]. Foxp3^+^ regulatory T cells (Treg) play a critical role in maintaining self-tolerance, and co-transfer of Treg with pathogenic effector cells can prevent autoimmune disease development [3]–[5]. Previous animal studies using Treg-based cell therapies demonstrated that this approach is efficacious in controlling alloimmune responses to organ and cell transplants [6]–[11].

Extensive pre-clinical animal studies have shown that Treg are crucial for controlling T1D development [12]–[15]. While the exact pathogenic mechanisms leading to T1D still remain largely unclear, two different hypotheses have been proposed. First, it has been hypothesized that the presence of a defective Treg population in T1D patients contributes to diabetes development [16]–[21]. In contrast, the second hypothesis suggests effector T cells (Teff) in T1D patients are more resistant to Treg suppression, which may contribute to onset of T1D [22], [23]. Recent studies, however, have found no difference in Treg frequency in peripheral blood in comparison with that in healthy controls [20], [24], [25], and it is also not clear if Teff population in all T1D patients are Treg-resistant. These uncertainties were further compounded by more recent findings that Treg from long-term T1D patients may retain their suppressive function while circulating in the peripheral blood, but this function is lost once Treg enter and reside in the pancreas [26]. In addition, there may be an altered population of Th1 vs. Th17 cells, suggesting dysregulation of both Treg and Teff in T1D patients.

Notwithstanding the complexity of Treg and their effects on Teff in T1D patients, Treg have great potential to be used as a novel cell-based treatment to restore self-tolerance and to treat T1D. Previous studies have shown that Treg from newly-onset T1D patients can be isolated and expanded *in vitro* to therapeutically-relevant levels [27]. Nevertheless, it is still unclear whether functionally potent Treg can also be expanded from patients with long-term T1D, and if a T1D patient's own Treg can be used to reduce the autoimmunity of T1D in autologous Treg transplantation studies. It is also not known whether such Treg can be used to prevent the rejection of allogeneic islet grafts. To address these questions either pre-clinically or clinically, it is important to take into consideration the fact that Treg are not a uniform population, consisting of phenotypically diverse subpopulations. It is unclear whether these subsets of Treg represent functionally distinct subsets, or if they exist due to intrinsic transcriptional plasticity [28]–[30].

Therefore, prior to using Treg in cell-based immunotherapies, it is important to examine the following questions: are the expanded Treg from patients with long-term T1D phenotypically different from that expanded from healthy subjects? How well can the Treg from those T1D patients be expanded *in vitro*? Can these expanded Treg retain potent functionality in suppressing Teff from both autologous and allogeneic subjects compared to Treg expanded from healthy subjects? Herein, we report the results from our studies in addressing these important questions. The findings from these studies should aid in designing preclinical and clinical approaches using *in vitro* expanded Treg, with the aim of helping patients with long-term T1D to restore immune tolerance that will prevent destroying insulin-producing cells.

## Results

### Altered CD45RA and CD45RO expression on Foxp3^+^ Treg in PBLs of T1D patients

**Figure 1:**
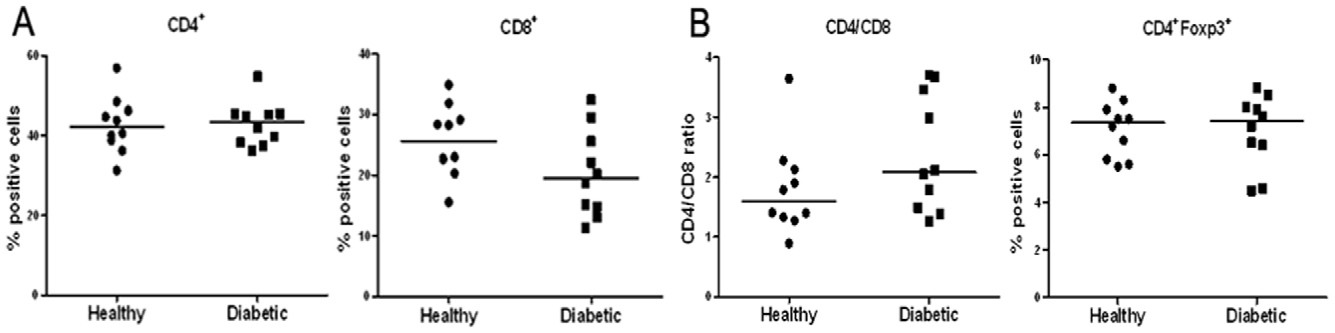
Comparable frequency of CD4^+^, CD8^+^ or Treg in PBLs of T1D patients and healthy subjects. PBMCs isolated from the whole blood of both diabetic patients and healthy controls were stained with indicated antibodies and analyzed by flow cytometry. (**A**) Percentage of CD4^+^ and CD8^+^ T cells in PBLs. (**B**) Ratios of CD4^+^ and CD8^+^ T cells (left) and the frequency of CD4^+^Foxp3^+^ Treg (right). The horizontal lines represent the median of all analyzed samples in each group.

We first analyzed the percentage of CD4^+^ and CD8^+^ T cells in PBLs from both patients with established T1D and healthy subjects. As shown in Table 1, Fig. 1A–B, both groups had comparable percentages and ratio of CD4^+^ and CD8^+^ T cells. In addition, consistent with previous findings [24], there was no difference in the frequency of CD4^+^Foxp3^+^ Treg in the PBLs between T1D and healthy subjects (Fig. 1B).

**Table 1:** T cell subset distribution in peripheral blood of healthy and diabetic subjects*.

Subject	Gender	Age	% CD4^+^ cells in lymphocyte	% CD8^+^ cells in lymphocyte	CD4/CD8 ratio	% CD4^+^Foxp3^+^ Treg
1	F	33	56.9	15.6	3.6	5.5
2	F	29	48.5	22.8	2.1	5.6
3	M	37	46.2	20.3	2.3	7.5
4	F	24	40.1	28.4	1.4	7.9
5	F	37	40.5	22.6	1.8	7.5
6	F	26	38.8	29.1	1.3	7.2
7	M	37	31.3	34.9	0.9	8.8
8	F	25	44.7	31.9	1.4	8.3
9	M	31	36.2	28.3	1.3	5.8
10	M	54	43.8	23	1.9	6.6
11	M	59	37.5	29.5	1.3	7.9
12	M	52	36.2	20.2	1.8	8.8
13	M	46	54.8	14.9	3.7	8
14	F	33	45.3	22.1	2	4.5
15	F	61	44.9	32.4	1.4	4.6
16	F	52	41.9	11.3	3.7	6.4
17	F	48	39.7	18.8	2.1	7.2
18	F	50	45.2	15.1	3	7.6
19	F	22	45.4	13.1	3.5	8.5
20	M	64	38.2	25.6	1.5	6.5

* Subjects 1–10 were healthy controls and subjects 11–20 were T1D patients.

To further compare T cell subset populations between the two subject groups, we examined the expression of CD45RA and CD45RO on T cells, representing naïve and memory T cells, respectively. In particular, we also analyzed the expression of CD45RA and CD45RO on CD4^+^Foxp3^+^ Treg. The results showed that the CD4^+^ T cells in PBLs of T1D subjects had less CD45RA^+^ (*p* = 0.0007) and more CD45RO^+^ (*p* = 0.0043) T cells than did healthy controls. In addition, the T1D subject PBLs also showed a lower frequency of CD45RA^+^CD8^+^ T cells than that of healthy controls, while they show a comparable frequency of CD45RO^+^CD8^+^ subset.

**Figure 2:**
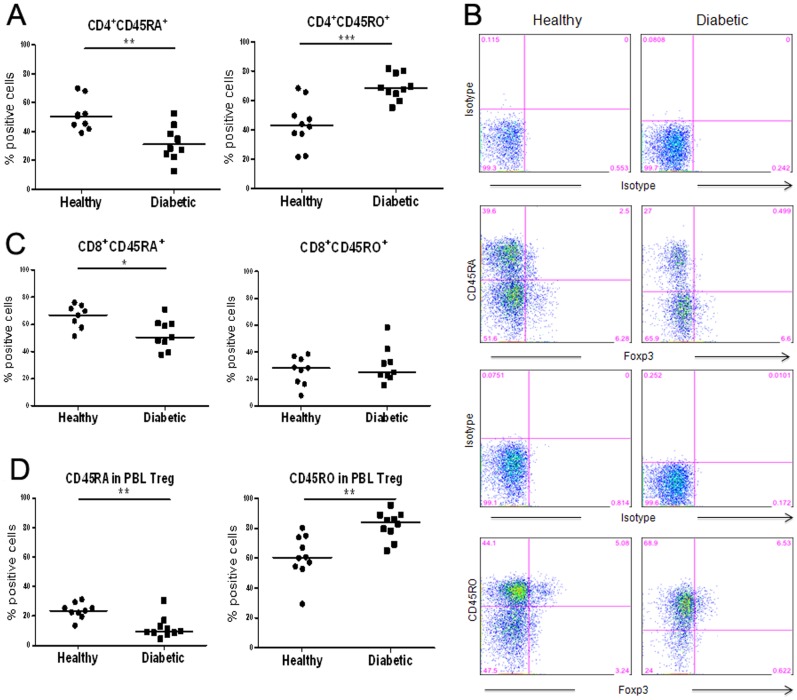
Altered populations of CD45RA or CD45RO-expressing Treg in T1D patients. PBMCs isolated from the whole blood of both diabetic patients and healthy controls were stained with indicated antibodies and analyzed by flow cytometry. (**A**) Percentage of CD45RA^+^ or CD45RO^+^ cells detected in the CD4^+^ or CD8^+^ T cells. (**B**) Percentage of CD45RA^+^ or CD45RO^+^ cells in CD4^+^Foxp3^+^ Treg. (**C**) Representative results from FACS analyses of CD45RA, CD45RO and Foxp3 expression in the CD4^+^ T cells in PBLs. The cells were electronically gated for FACS analyses. *p<0.05, **p<0.01, ***p<0.001.

Within the CD4^+^ T cells in PBLs, we further analyzed the expression of CD45RA and CD45RO on CD4^+^Foxp3^+^ Treg. Similar to CD4^+^ T cells, less Treg in the PBLs of T1D subjects express CD45RA than healthy controls (*p* = 0.002)(Fig. 2B–C). In addition, more Foxp3^+^ Treg in the CD4^+^ T cells of T1D subjects express CD45RO than in healthy controls (*p* = 0.0011)(Fig. 2B–C). Further analyses showed that the observed difference in cell phenotype is independent of the age of subjects in the two cohorts (data not shown). Overall, these data support the conclusion that a larger percentage of not only CD4^+^ T cells but also CD4^+^Foxp3^+^ Treg in PBLs from T1D subjects than those from healthy controls express the CD45RO^+^ memory cell phenotype, and thus are likely antigen-experienced cells.

### Treg from both diabetic and healthy subjects can be expanded in vitro equally well with high purity

**Figure 3:**
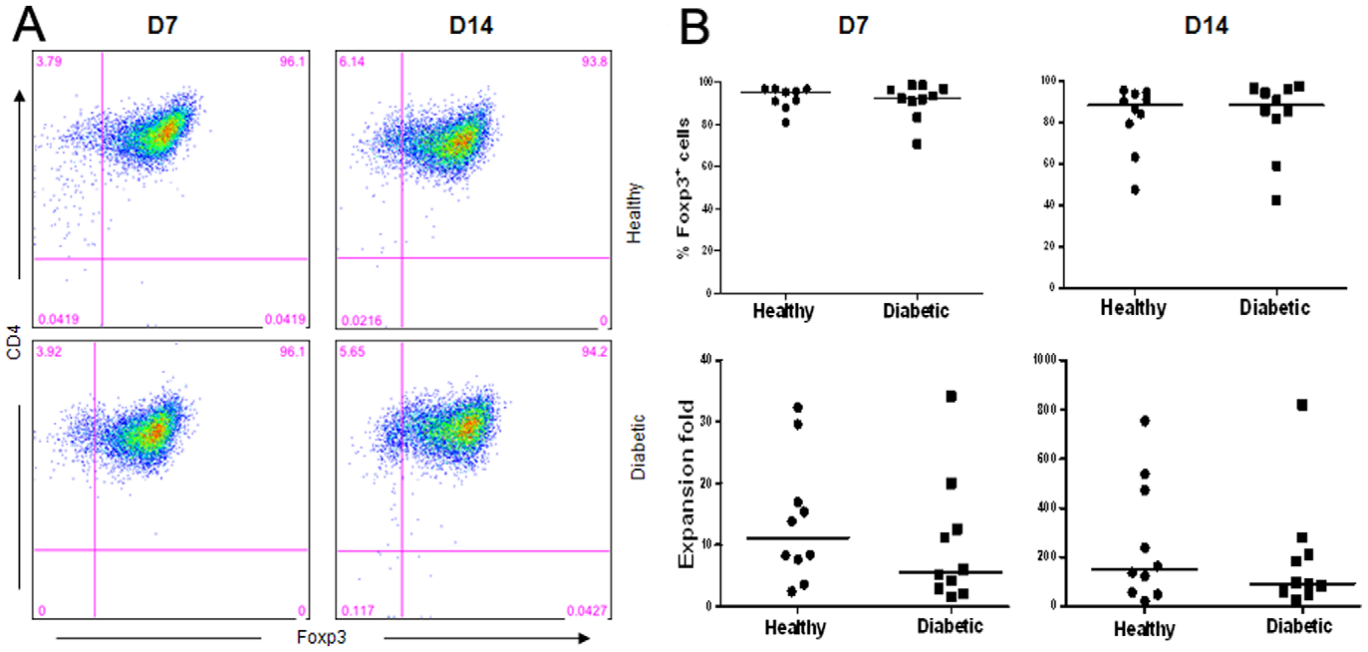
Both diabetic and healthy Treg can be expanded with comparable purity and yield *in vitro*. CD4^+^CD25^+^CD127^−/lo^ Treg, electronically sorted using FACS from diabetic patients and healthy subjects, were activated in vitro using anti-CD3/anti-CD28-coated beads plus recombinant human IL-2. Expanded Treg were stained with CD4, CD25 and Foxp3 antibodies and analyzed by FACS on day 7 (D7) and day 14 (D14) following activation. (**A**) FACS analyses of CD4 and Foxp3 expression of expanded Treg on D7 and D14. The representative results were from cells obtained from one individual of each subject group. (**B**) Collective analyses of the purity and expansion fold of expanded Treg cells in each group on D7 and D14.

Next, we determined, compared to healthy controls, how well Treg isolated from PBLs of T1D patients with established long-term disease can be expanded *in vitro*. Previous studies showed that the CD4^+^CD25^+^CD127^−/lo^ T cells contain the greatest population of Foxp3^+^ Treg [21]. Therefore, we isolated CD4^+^CD25^+^CD127^−/lo^ T cells from PBLs of T1D or healthy subjects to obtain a highly purified Foxp3^+^ Treg population. Similar purity and cell yield of sorted Treg were obtained from both subject groups on day 0 (mean: 97.6±1.8%, range: 93.3%–99.9%)(Table 2). The purified Treg were then activated and expanded *in vitro*. During the culture period, there was no significant difference in the purity of cultured Treg isolated from T1D or healthy subjects on day 7 and day 14 (Fig. 3A–B). Furthermore, the purified Treg from both groups could be expanded equally well *in vitro* (Fig. 3B).

**Table 2:** Expanded CD4^+^CD25^+^CD127^lo/−^ T cells from healthy controls and T1D patients are highly enriched Foxp3^+^ Treg*.

			Purity (%) of initially-sorted cells	Purity (%) of expanded CD4^+^Foxp3^+^ cells	
Subject	Gender	Age	CD4^+^CD25^+^CD127^lo/−^	Day 7	Day 14
1	F	33	96.4	96.6	94.7
2	F	29	95.1	95.1	93.7
3	M	37	93.3	82.1	63.2
4	F	24	99.5	91.3	90.3
5	F	37	95.7	90.8	86.3
6	F	26	97.1	96.6	95.6
7	M	37	99.5	96.6	91.1
8	F	25	99.2	95.4	84.1
9	M	31	97.9	80.8	47.6
10	M	54	97.7	87.9	81.1
11	M	59	95.7	93.3	85.7
12	M	52	95.7	90.8	81.9
13	M	46	99.1	91.8	90.9
14	F	33	99.3	98.3	96.4
15	F	61	99	96.1	94.2
16	F	52	99.9	98.3	97.5
17	F	48	98.4	83.2	59.1
18	F	50	97.6	96.6	95.9
19	F	22	98.6	92	85.5
20	M	64	98.1	70.6	42.5

* Subject 1–10 were healthy controls and subject 11–20 were T1D patients.

### In vitro expansion leads to decreased expression of Helios on Treg from diabetic patients but not from healthy controls

**Figure 4:**
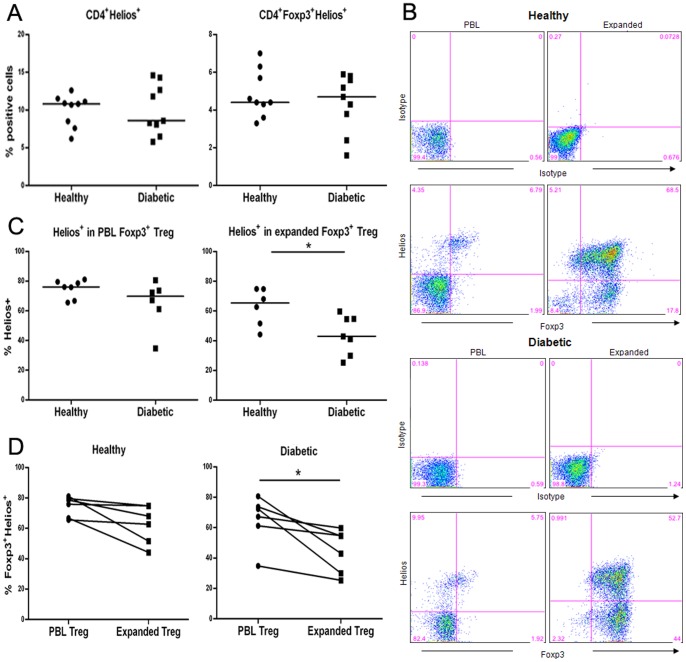
*In vitro* expansion leads to decreased expression of Helios on T1D patient but not healthy subject Treg. PBMCs and expanded Treg were stained with indicated antibodies and analyzed by FACS. (**A**) Percentage of Helios^+^ cells in total CD4^+^ T cells (CD4^+^Helios^+^) or in Foxp3^+^CD4^+^ T cells (CD4^+^Foxp3^+^Helios^+^) in PBLs of T1D patients or healthy controls. (**B**) FACS analyses of Helios and Foxp3 expression in PBLs and expanded Treg on D14 from one representative individual of each subject group. The cells were electronically gated on CD4^+^ T cell population. (**C**) Collective analyses of the percentage of Helios^+^ cell frequency in PBLs or expanded Foxp3^+^ Treg on D14 in each subject group. (**D**) Paired comparison analyses of Helios expression on Foxp3^+^ Treg in PBLs vs. its expression on expanded Foxp3^+^ Treg in each subject group.

Helios, an Ikaros transcription factor family member, has been reported as a marker that distinguishes thymically-derived nTreg from peripherally-induced Treg (iTreg) and/or as a marker for T-cell activation and proliferation [32]–[34]. We first compared the expression of Helios on non-cultured CD4^+^ T cells and CD4^+^Foxp3^+^ Treg in PBLs of T1D and healthy subjects and in the expanded Foxp3^+^ Treg from their PBLs. Our analyses on non-cultured CD4^+^ T cells showed comparable percentages of Helios^+^ or Helios^+^Foxp3^+^ CD4^+^ T cells detected in T1D subjects with those detected in healthy controls (Fig. 4A). Next, we assessed Helios expression within the CD4^+^Foxp3^+^ Treg population in PBLs, and found no difference of Helios expression in these Treg between the two subject groups (Fig. 4B–C). In addition, there was also no difference in the Helios expression on Foxp3^+^ Treg in CD4^+^CD25^+^CD127^−/lo^ PBLs from T1D patients or healthy subjects (data not shown).

We then compared Helios expression on the expanded Treg from PBLs of T1D or healthy subjects. After expansion *in vitro*, fewer Treg from T1D subjects than from healthy controls expressed Helios (*p* = 0.03)(Fig. 4B–C). The reduction of Helios expression was further confirmed from the paired comparison analyses of Helios expression on Foxp3^+^ Treg in PBLs versus its expression in the expanded Foxp3^+^ Treg from the same subjects. These results demonstrate that a significantly decreased percentage of Treg from T1D but not from healthy subjects expressed Helios during expansion *in vitro* (*p* = 0.02, Fig. 4D).

Altogether, these results suggest that the loss of Helios expression on Treg from diabetic patients may have occurred during the culture and expansion of the cells in vitro.

### Analyses of additional markers that may be associated with Treg lineage or function

**Figure 5:**
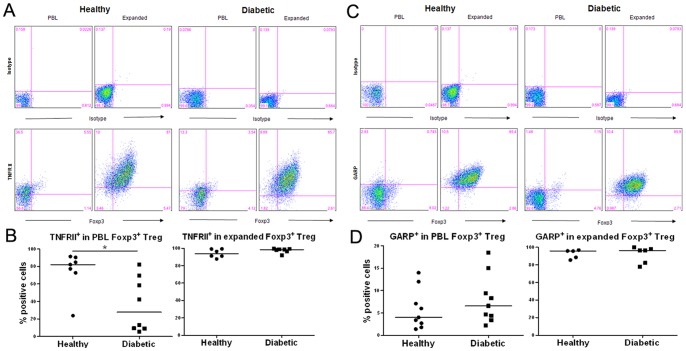
TNFRII and GARP expression on Foxp3^+^ Treg. PBMCs and expanded Treg were stained with indicated antibodies and analyzed by FACS. FACS analyses of (**A**) TNFRII, (**C**) GARP expression on Foxp3^+^Treg in PBLs and expanded Treg from one representative individual of each subject group. Cells were electronically gated on CD4^+^ T cell population. Collective analyses of the percentage of (**B**) TNFRII^+^ or (**D**) GARP^+^ cell frequency in PBLs or expanded Foxp3^+^ Treg.

We also analyzed the expression of additional markers, including TNFRII and GARP, which can be found on CD4^+^Foxp3^+^ Treg [35], [36]. Our results showed that, within the CD4^+^Foxp3^+^ Treg population in PBLs, a significantly lower frequency of non-cultured Treg from T1D subjects expressed TNFRII compared to healthy controls (*p* = 0.01)(Fig. 5A–B). After expansion *in vitro*, a comparable percentage of TNFRII expression was found on the expanded Treg derived from T1D or healthy subjects. Compared to TNFRII, our results showed no difference in the expression of GARP on CD4^+^Foxp3^+^ Treg in PBLs or on expanded Treg from both subject groups (Fig. 5C–D).

Altogether, these results indicate that Treg from T1D patients increased their expression of TNFRII during *in vitro* expansion, without changing the expression pattern of GARP on activated and expanded Treg, compared to those of healthy controls.

### T1D patients contain an increased population of Th1 cells and a comparable Th17 cells in PBLs, compared to healthy controls

**Figure 6:**
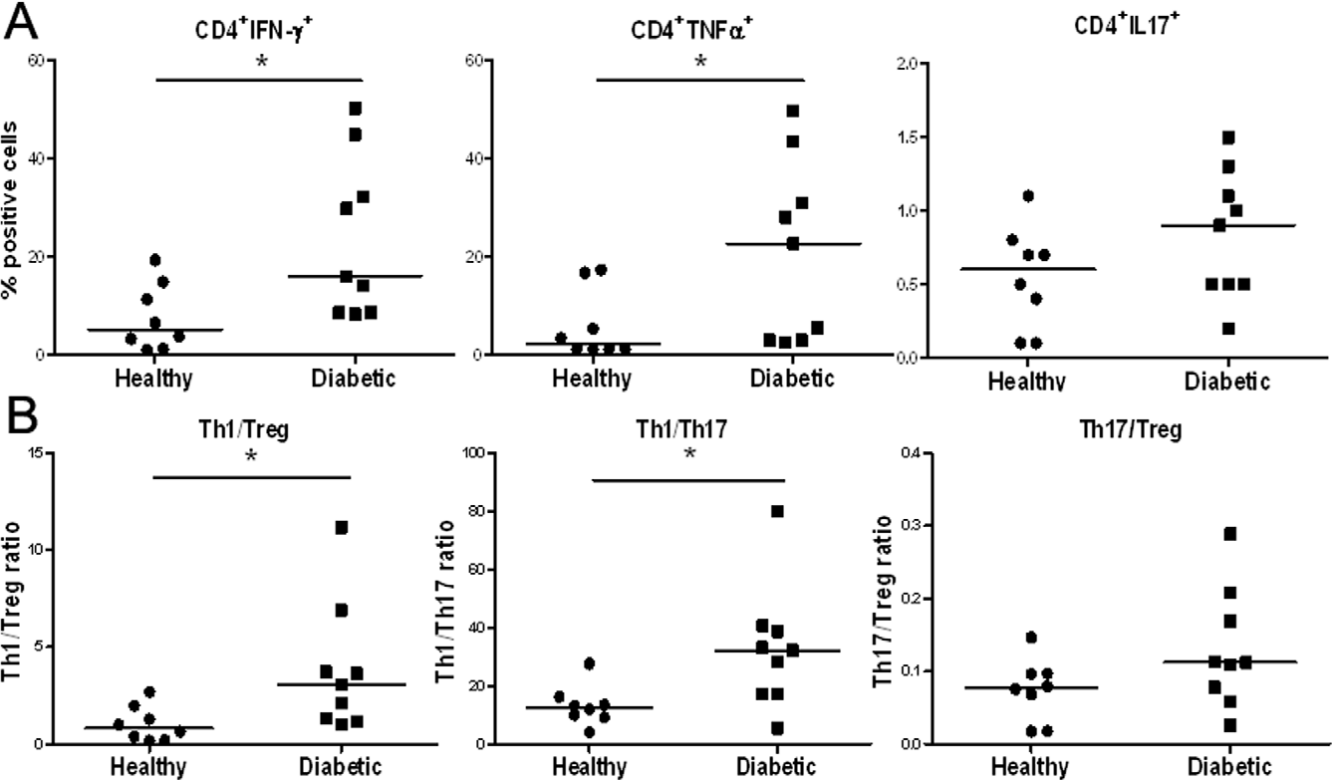
Increased IFN-γ or TNF-α-producing Th1 cells in T1D patients. PBMCs were stained with indicated antibodies following activation with PMA/ionomycin for 5 h in vitro and analyzed by FACS. (**A**) Percentage of IFN-γ^+^, TNF-α^+^,or IL17^+^ cells present in CD4^+^ T cells of PBLs from T1D patients or healthy controls. (**B**) The ratios of Th1/Treg, Th1/Th17, and Th17/Treg in CD4^+^ T cells from PBLs of diabetic patients or healthy controls.

Although T1D is a T-cell-mediated disease, the specific pathogenic mechanisms leading to T1D remain largely unclear. In particular, further studies are needed to elucidate the relative roles of Th1 and Th17 cells versus Treg during the onset and further development of T1D in human patients [37]. One hypothesis suggests that imbalanced pro-inflammatory T cell subsets (including Th1 and Th17 cells) versus Treg in T1D patients may contribute to T1D pathogenesis. To begin addressing this issue, we performed intracellular staining analyses of IFN-γ, TNF-α, IL17, or Foxp3 on PBLs of T1D and healthy subjects. The results showed an elevated IFN-γ (*p* = 0.02) and TNF-α (*p* = 0.03) expression in CD4^+^ T cells in PBLs of T1D subjects than those of healthy controls (Fig. 6A). In comparison, only a small percentage of IL17-expressing CD4^+^ cells were present in both T1D subjects and healthy controls, and there was no difference between the two groups (Fig. 6A). Further analyses showed that the observed difference in cell phenotype is independent of the age of subjects in the two cohorts (data not shown). Therefore, a significantly increased Th1 but not Th17 cell population is present in the PBLs of T1D patients compared to those of healthy controls.

Based on the percentage of cells positively stained for IFN-γ, IL17, or Foxp3, we further calculated the ratio of Th1/Treg, Th1/Th17, or Th17/Treg in CD4^+^ T cells of PBLs from both subject groups. The ratios of IFN-γ^+^Th1 cells to Foxp3^+^Treg or IL17^+^Th17 cells were increased in T1D patients (*p*<0.05, Fig. 6B). On the other hand, the ratio of Th17 cells to Treg was comparable to that in healthy controls. These results demonstrate the presence of an imbalanced CD4^+^ T cell subsets with significantly increased Th1 cells in PBLs of T1D patients than in healthy controls. In comparison, no significant difference exists for the Th17/Treg ratio between the two groups.

These results support the novel hypothesis that, compared to healthy subjects, T1D patients contain a significantly increased population of pathogenic Th1 cells such that their unchanged normal level of Treg may not be sufficient to effectively suppress islet destruction by the increased number of pathogenic Th1 cells.

### More Treg from PBLs of T1D subjects than healthy subjects express IFN-γ or TNF-α

**Figure 7:**
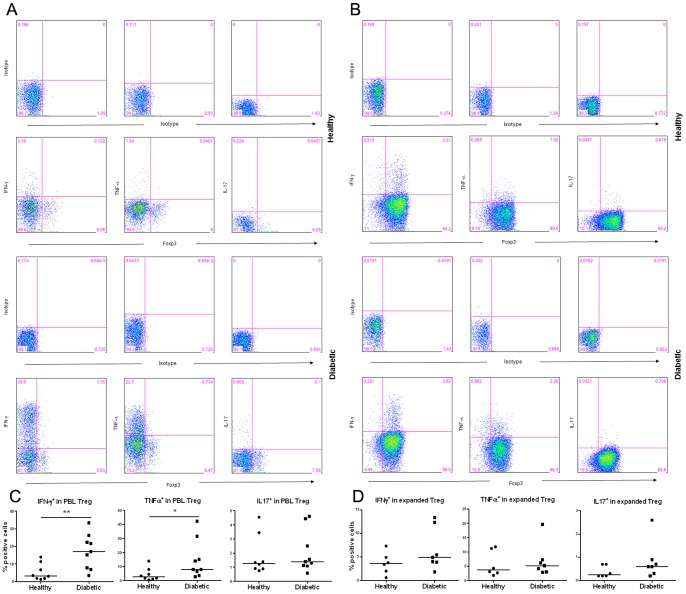
Expression of IFN-γ, TNF-α, IL17 in Treg of PBLs or in expanded Treg. Cells from PBMCs and expanded Treg on Day 14 after anti-CD3/CD28 activation were stained with indicated antibodies following stimulation with PMA/ionomycin for 5 h and analyzed by FACS. (**A**–**B**) IFN-γ, TNF-α, and IL17 expression in Foxp3^+^Treg of (**A**) PBLs and (**B**) expanded Treg from one representative individual of each subject group. Cells were electronically gated on CD4^+^ T cell population. **(C**–**D**) Collective analyses of the percentage of IFN-γ^+^, TNF-α^+^, or IL17^+^ in (**C**) Foxp3^+^ Treg of PBLs or (**D**) expanded Foxp3^+^ Treg.

Previous studies showed that some human Foxp3^+^ Treg might also express IFN-γ [25]. We first examined whether there would be differences in IFN-γ expression in Treg from CD4^+^ PBLs of T1D subjects versus those from healthy controls. In addition, we also examined whether Treg may also express another Th1 cytokine TNF-α. We found that there were more IFN-γ^+^ or TNF-α^+^ (*p* = 0.003) cells in the CD4^+^Foxp3^+^ Treg from PBLs of T1D subjects than in those of healthy controls (Fig. 7A and 7C). A small and comparable percentage of the CD4^+^Foxp3^+^ Treg in PBLs from both T1D and healthy subjects expressed IL17. Further analyses showed that the observed difference in cell phenotype is independent of the age of subjects in the two cohorts (data not shown).

We then analyzed whether expanded Treg may continue expressing IFN-γ, TNF-α or IL17. As shown in Fig. 7B and 7D, a comparable percentage of expanded Treg from both groups expressed IFN-γ, TNF-α, and IL17. These results also showed that, after *in vitro* expansion, T1D patients' Treg significantly reduced their expression of these three pro-inflammatory cytokines to a level comparable to those found in Treg from healthy controls.

### Comparable suppressive functionality of expanded Treg from both diabetic and healthy subjects

**Figure 8:**
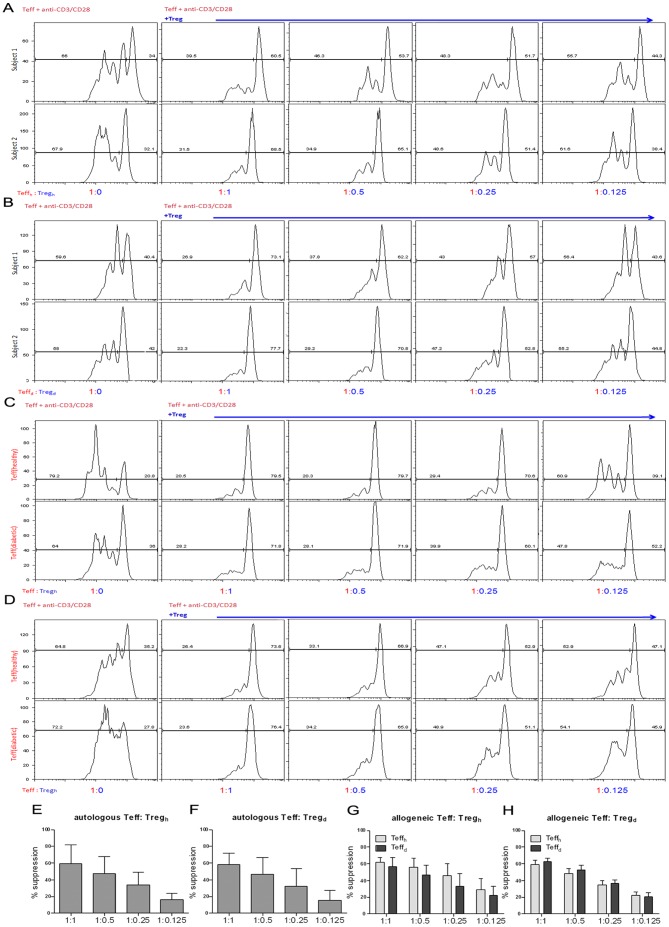
Treg expanded from diabetic patients or healthy subjects have comparable potent suppressive function. CFSE-labeled PBMCs were co-cultured with expanded Treg from the same donor or allogeneic donors at various ratios (Teff/Treg = 1∶0, 1∶1, 1∶0.5, 1∶0.25 or 1∶0.125). The cells were stimulated with soluble anti-CD3/anti-CD28 antibodies for 3 days, and the proliferation of CFSE-labeled CD8^+^ T cells in PBMCs was analyzed by FACS. (**A**–**B**) Results from functional analyses of co-culture of expanded Treg with autologous PBMCs from two representative healthy subjects (**A**) or diabetic patients (**B**) from at least 8 different subjects for each group. (**C**–**D**) Results from functional analyses of co-culture of expanded Treg with allogeneic PBMCs from either healthy subjects or diabetic patients. The Treg were from one representative healthy subject (**C**) or T1D patient (**D**). (**E–F**) The % suppression as a function of Teff/Treg ratio for Treg from either healthy controls (**E**) or diabetic patients (**F**) using autologous Teff. (**G–H**) The % suppression as a function of Teff/Treg ratio for Treg from either healthy controls (**G**) or diabetic patients (**H**) using allogeneic Teff from either healthy controls or diabetic patients.

To compare the function of *in vitro* expanded Treg from the two subject groups, we first examined whether the expanded Treg may suppress the proliferation of autologous CD8^+^ Teff in PBLs from the same subject. Results from two representative subjects of either T1D patient or healthy controls are shown in Fig. 8A–B. Following a 3-day incubation of autologous PBLs with expanded Treg plus anti-CD3/CD28, the proliferation of CFSE-labeled CD8^+^ Teff cells in PBLs was analyzed at the end of the assay. The results demonstrated that the suppressive function of the expanded Treg was dose-dependent. When compared at varied Teff/Treg ratios, the expanded Treg from both T1D and healthy subjects were able to suppress autologous Teff equally well (Fig. A and E, B and F). In addition, the expanded Treg from both subject groups were still able to suppress Teff proliferation at a 1∶0.125 Teff∶Treg ratio. Therefore, expanded Treg from T1D patients or healthy subjects have comparable function in suppressing autologous Teff cells.

We then determined whether the expanded Treg were able to suppress allogeneic Teff from either healthy or T1D subjects. We performed a series of criss-cross suppression assays in parallel, including co-culture of expanded Treg from T1D subjects (Treg_diabetic_) with Teff from allogeneic T1D subjects or healthy controls. The same assay was also performed using expanded Treg from healthy subjects (Treg_healthy_) with Teff from allogeneic T1D subjects or healthy controls. As shown in Fig. 8C–D, both the expanded Treg_healthy_ and Treg_diabetic_ were able to suppress allogeneic Teff from either healthy or T1D subjects. In comparison, the expanded Treg from either cohort were also able to suppress allogeneic Teff from both healthy and T1D subjects equally well (Fig. 8G–H). These results further showed that the suppressive function of expanded Treg from both groups was dose dependent, and they still have suppressive effect on allogeneic Teff from healthy or T1D subjects at a 1∶0.125 Teff∶Treg ratio.

Overall, these results demonstrate that *in vitro* expanded Treg from diabetic patients function as potent suppressors as well as those from healthy controls. In particular, these expanded Treg are able to suppress Teff from not only autologous subjects, but also from allogeneic healthy or T1D subjects.

## Discussion

In this study, we demonstrated that: (1) PBLs from patients with established long-term (>15 yr) T1D contained an increased population of IFN-γ and/or TNF-α-producing Th1 cells and comparable Th17 cells and Treg compared to those in healthy subjects. (2) A significantly increased population of not only CD4^+^ T cells but also CD4^+^Foxp3^+^ Treg in PBLs from T1D patients than those from healthy subjects expressed CD45RO^+^ memory cell phenotype and are likely antigen-experienced cells. (3) Treg from both T1D patients and healthy subjects were expanded *in vitro* equally well with high purity, and *in vitro* expansion led to decreased Helios expression on Treg from T1D patients but not from healthy subjects. (4) More Treg from PBLs of T1D patients than from healthy subjects expressed IFN-γ or TNF-α. After *in vitro* expansion, the expanded Treg from T1D patients reduced expression of these pro-inflammatory cytokines to a level comparable to those found in expanded Treg from healthy controls. (5) Expanded Treg from both subject groups have comparable function in suppressing Teff cells from either autologous or allogeneic subjects.

The pathogenesis of T1D in human patients remains largely unresolved. One hypothesis suggests a defect in Treg number may play an important role in the onset of T1D [38]. However, our results and those from several recent studies in humans have demonstrated that the frequency of Treg in peripheral blood was comparable between T1D patients and healthy subjects [20], [24], [25]. In addition, Foxp3^+^ Treg in humans represent a heterogeneous population of cells expressing CD45RA^+^ naïve Treg or CD45RO^+^ memory Treg [24], [39], [40]. Our results demonstrated that, for the subjects involved in our analyses, the T1D patients have significantly less CD45RA^+^ Treg and more CD45RO^+^ Treg than healthy subjects. These results suggest that more Treg in T1D patients than in healthy subjects may have been previously activated and are antigen-experienced memory cells. However, these results do not exclude the possibility that, if a larger number of subjects are included in the studies, a wider age difference between T1D patients and healthy subjects may have a significant impact on the expression of CD45RA and CD45RO on Treg in PBLs from the subjects of the two cohorts. Although previous studies suggested that CD45RA^+^ Treg may expand better than CD45RO^+^ counterparts [27], our results showed that Treg isolated from T1D patients and healthy subjects expand equally well *in vitro.* Therefore, a sufficient number of Treg can be expanded from patients with long-term established T1D such that they may be used for autologous Treg-based cell therapy to treat T1D and/or prevent islet graft rejection.

Recent studies suggest that differential expression of the transcription factor Helios in Treg may be used to distinguish nTreg from iTreg [33]. Alternatively, Helios can be used as a marker for T-cell activation and proliferation [34], and can be expressed in iTreg after antigen exposure [41]. In this study, we found no difference in Helios expression in Treg from PBLs of T1D patients and healthy subjects; however, our results also found that, after expansion *in vitro,* fewer expanded Treg from T1D patients than those from healthy subjects expressed Helios. Therefore, more Treg from T1D patients than from healthy subjects tend to lose Helios expression during expansion *in vitro*. These results suggest that nTreg from T1D patients may not proliferate as fast as the peripherally-induced iTreg. Alternatively, if the Helios is considered as a T cell activation marker, the activation status of expanded Treg may not be the same between the two subject groups. In addition, the role of Helios in Treg still remains unclear. Recent work showed that Helios expression may precede Foxp3 expression after antigen stimulation, but its expression becomes less stable in the absence of continuous antigen exposure [34]. Based on our findings and those of others, there is the possibility that Treg from long-term T1D patients may be less responsive to activation and require stronger stimulation through TCR to obtain a large number of Treg than those from healthy controls.

A well-balanced population between Treg and Th1 or Th17 effector cell subsets is crucial for maintaining immune cell homeostasis and tolerance. Our results showed a significantly increased IFN-γ^+^ and/or TNF-α^+^ Th1 cells but not IL-17^+^ Th17 cells in the PBLs of T1D patients compared to those of healthy subjects. These results are consistent with the hypothesis that T1D is mainly a Th1-mediated autoimmune disease. On the other hand, recent findings showed that the immune balance is shifted toward a Th17 environment in the pancreatic-draining lymph nodes of long-term T1D patients but not in their peripheral blood [26]. These results suggest that both Th1 and Th17 cells may play pathogenic roles in T1D, and their roles may be dependent on tissue location during T1D development. More extensive studies on natural history of T1D would help further reveal the individual roles of various Teff cells in T1D.

Previous studies showed that both Foxp3^+^ and Foxp3^−^ Treg can express IFN-γ [25], [42], [43]. These IFN-γ^+^ Treg, considered as iTreg instead of nTreg, also have potent suppressive function that can suppress target Teff cells. In this study, our results showed that a significantly increased IFN-γ^+^ or TNF-α^+^ cells are present in the CD4^+^Foxp3^+^ Treg population in PBLs of T1D patients than of healthy subjects. These findings suggest that there are relatively more iTreg in the peripheral blood of patients with long-term T1D than in healthy subjects, although comparable frequency of Foxp3^+^ Treg is found in PBLs from both groups. After expansion *in vitro*, however, a comparable percentage of Treg expanded from PBLs of both subject groups expressed IFN-γ or TNF-α. The reason for reduced IFN-γ^+^ or TNF-α^+^ cells in Treg expanded from T1D patients is unclear. One possibility is that these Treg subsets from T1D patients might be less stable than those from healthy subjects, or they tend to lose their proliferative potential more easily during expansion *in vitro.* Our previous studies in mice have shown that the function of autoantigen-specific Foxp3^−^ Treg is dependent on IFN-γ [42]. It is unclear whether IFN-γ may also play a similar regulatory role in T1D patients or healthy subjects. Further studies will be needed to clarify the role and function of Th1 cytokine-expressing Treg in the induction or maintenance of immune tolerance in humans.

In conclusion, to develop cell-based immunotherapy, it is desirable to use autologous Treg to induce immune tolerance. Thus, the use of autologous Treg following large-scale expansion while retaining potent regulatory function would be an ideal approach to treat T1D or to prevent islet graft rejection. Our results showed that expanded Treg from patients with long-term T1D are able to effectively suppress Teff from not only autologous subjects but also from allogeneic healthy or T1D subjects. In addition, the expanded Treg from T1D patients are as potent suppressors as those from healthy controls. These results suggest that expanded Treg may be used *in vivo* to restore immune tolerance in T1D patients or to prevent allogeneic islet graft rejection. While this forms the basis for a promising therapeutic approach, other recent work showed that Treg may lose their immune suppressive activity once they reside within the site of autoinflammatory drainage, although they retain a suppressive function when circulating in the periphery [26]. Therefore, further pre-clinical *in vivo* studies are necessary to determine whether the expanded human Treg are indeed able to restore immune tolerance as expected, or whether they would have a better protective effect in preventing islet grafts transplanted into alternative sites.

## Materials and Methods

### Ethics statement and subject collection

We collected 10 adults with established long-term T1D (4 men/6 women, mean age 48.7±12.8, range 22–64, with disease duration >15 years) and 10 non-diabetic healthy control subjects (4 men/6 women, mean age 33.3±8.8, range 24–54) in our study (Table 3). All T1D patients were diagnosed according to American Diabetes Association criteria [31]. Sample acquisition and the procedures of the current study were specifically approved by the Institutional Review Boards (IRB) at the City of Hope in accordance with the Declaration of Helsinki. All donors have signed the informed consent forms.

**Table 3:** Demographics of healthy and diabetic subjects.

	Healthy	Diabetic
# of subjects	10	10
Gender (male∶female)	4∶6	4∶6
Age: Mean (range)	33.3(24–54)	48.7(22–64)
C-peptide	—	<0.1–0.18 ng/ml
Disease duration	—	>15 yrs

### Isolation and in vitro expansion of Treg

Fresh peripheral blood was collected in sodium-heparinized vacutainer tubes {Beckton Dickinson (BD), Franklin Lakes, NJ}, and red blood cells were removed by HESPAN sedimentation (B. Braun Medical, Irvine, CA). Peripheral blood mononuclear cells (PBMCs) were isolated by Ficoll-Paque gradient centrifugation (GE Healthcare, Piscataway, NJ), and stained with antibodies against CD4, CD25, and CD127 (BD Biosciences, San Diego, CA) [27]. CD4^+^CD25^+^CD127^−/lo^ cells were sorted by a FACSAria III cell sorter. For some subjects, CD4^+^ T cells were enriched with a human CD4^+^ lymphocyte enrichment kit (BD) prior to staining. Sorted cells were cultured in X-VIVO 15 (Lonza, Walkersville, MD) media containing 10% human heat-inactivated AB serum (Valley Biomedical, Winchester, VA) plus anti-CD3/CD28 Dynabeads ((Invitrogen; Carlsbad, CA) at a 1∶1 cell-to-bead ratio. At day 2, recombinant huIL-2 was added (300 units/ml, Peprotech, Rocky Hill, NJ). Fresh media and IL-2 were added at days 5, 7, 9, and 12. At day 9, cells were re-stimulated with anti-CD3/CD28 beads. At day 14, beads were removed and cells cultured for another 2 days prior to suppression assay. At days 7 and 14, the cultured cells were stained for CD4, CD25, and Foxp3.

### Flow cytometry and intracellular staining

The phenotype of freshly isolated PBMCs and expanded Treg were determined by staining with indicated antibodies and analyzed by FACSCanto II (BD). Antibodies to human CD4, CD8, CD25, CD127, TNFRII/CD120b, GARP, CD45RA and CD45RO were from BD, and Foxp3 and Helios from BioLegend.

Intracellular staining for IFN-γ, TNF-α, IL-17, Foxp3, and Helios was performed after staining for other cell surface markers. Fresh PBMCs and expanded Treg were activated with phorbol myristate acetate (PMA) (50 ng/ml) and ionomycin (500 ng/ml)(Sigma, Saint Louis, MO) plus GolgiStop (2 µl/ml, BD) for 5 h. Permeabilization was performed using the Foxp3 Fix/Perm Buffer kit (BioLegend). FACS data were analyzed using Flowjo software (Treestar, Ashland, OR).

### In vitro suppression assay

Suppressive function of expanded Treg was determined using PBMCs labeled with carboxyfluorescein diacetate succinimidyl ester (CFSE, Invitrogen/Molecular Probes, Eugene, OR) as targets. CFSE-labeled autologous or allogenic PBMCs (1×10^5^) were incubated with various ratios of Treg (1∶1, 1∶0.5, 1∶0.25, 1∶0.125 and 1∶0). Soluble anti-CD3 (5 µg/ml) and anti-CD28 (5 µg/ml) were used to stimulate cells. At day 3, cells were harvested and stained with anti-CD8 to assess cell proliferation. Electronically-gated CFSE-labeled CD8^+^ Teff cells were analyzed for their proliferation with varied Treg ratios. To further evaluate the function of expanded Treg, percentage of suppression was calculated using the following formula: (% CFSE-labeled CD8^+^ Teff in PBL having at least one cell division – % Treg-co-cultured CFSE-labeled CD8^+^ Teff in PBL having at least one cell division)/(% CFSE-labeled CD8^+^ Teff in PBL having at least one cell division)×100.

### Statistical analysis

The Mann-Whitney *U* test or a paired *t* test was used for comparisons between subject groups. A Spearman's test was used to identify correlations of the results for all applicable panels. The calculated *p*<0.05 was considered statistically significant. Analyses were performed using Graphpad Prism software (GraphPad Software Inc, San Diego, CA).
